# Regular extra-virgin olive oil intake independently associates with lower abdominal obesity

**DOI:** 10.3389/fnut.2025.1645230

**Published:** 2025-09-11

**Authors:** Carlo De Matteis, Lucilla Crudele, Ersilia Di Buduo, Salvatore Cantatore, Fabio Novielli, Silvia Cultrera, Angela Fulvia Tricase, Maria Arconzo, Marilina Florio, Raffaella Maria Gadaleta, Elena Piccinin, Marica Cariello, Antonio Moschetta

**Affiliations:** ^1^Department of Interdisciplinary Medicine, University of Bari “Aldo Moro”, Bari, Italy; ^2^INBB National Institute for Biostructure and Biosystems, Rome, Italy; ^3^Department of Translational Biomedicine and Neuroscience (DiBraiN), University of Bari “Aldo Moro”, Bari, Italy

**Keywords:** extra-virgin olive oil, Mediterranean diet, abdominal obesity, nutrition, waist circumference

## Abstract

**Background:**

Abdominal obesity is a major global health burden, driving risk for cardiovascular disease, type 2 diabetes, and cancer. The Mediterranean Diet (MedDiet), recognized for its cardiometabolic benefits, emphasizes Extra-Virgin Olive Oil (EVOO) as a primary fat source. We previously validated the Chrono Med Diet Score (CMDS), an index integrating dietary quality and chrono-nutritional principles, and demonstrated its associations with abdominal adiposity and cancer incidence. Although EVOO is central to the MedDiet, mechanisms related to its specific contributions to metabolic health remain partial. In the present study, we investigated the relationship between consistent EVOO intake frequency, MedDiet adherence (CMDS), and anthropometric outcomes.

**Methods:**

We analyzed data from 16,273 adults (46.5% male) who completed the CMDS-based online survey since April 2023. Data included age, sex, height, weight, waist circumference (WC), Body Mass Index (BMI) and dietary/lifestyle information. EVOO intake frequency was categorized as: sporadic (<3 days/week), frequent (≥3 but <6 days/week), or regular (≥6 days/week), based on ~25 g/day (~2 tablespoons). Statistical analyses included Student’s t-tests, ANOVA with Bonferroni correction, mediation analysis, and multivariable logistic regression adjusting for confounders.

**Results:**

Significant sex differences were observed in age, BMI, WC, and CMDS. Participants with regular EVOO intake were significantly older (55.9 ± 8.1 years) than sporadic (53.9 ± 7.1) and frequent (54.1 ± 7.7) consumers (*p* = 0.0019) yet showed more favorable anthropometrics. Compared to sporadic intake, regular intake was associated with significantly lower BMI (24.7 ± 3.0 vs. 26.6 ± 2.9, *p* < 0.001) and WC (89.1 ± 6.7 cm vs. 99.4 ± 9.1 cm, *p* < 0.0001), with consistent results across sexes (*p* < 0.0001 for both). Mediation analysis revealed that EVOO’s effect on WC was significantly mediated by CMDS (*β* = −0.83, *p* < 0.0001), accounting for 61.9% of the total effect. A direct association also persisted after adjusting for CMDS (*β* = −0.59, *p* < 0.0001). In logistic regression, non-regular EVOO intake was associated with substantially higher odds of abdominal obesity (Odds Ratio 5.1; 95% Confidence Interval: 3.3–6.8; *p* < 0.0001).

**Conclusion:**

In this large cohort, regular EVOO consumption, while defining higher CMDS adherence, is independently associated with lower BMI and WC. EVOO exerts a dual role in metabolic health, both mediating and independently enhancing the relationship between chrono-Mediterranean diet adherence and reduced abdominal obesity. Non-regular EVOO intake emerges as a strong risk factor for visceral adiposity, irrespective of overall diet quality.

## 1 Introduction

Abdominal obesity, characterized by excess fat accumulation around abdominal organs, is a primary driver of Metabolic syndrome (MetS) and is strongly associated with increased risks of cardiovascular disease, type 2 diabetes, cancers, and overall mortality (1–4). Abdominal fat is metabolically active, secreting inflammatory cytokines that contribute to systemic low-grade chronic inflammation and insulin resistance, further perpetuating metabolic dysfunction (5, 6). Consequently, identifying effective lifestyle strategies, to mitigate and reduce abdominal obesity, particularly dietary interventions, is a critical public health priority (7). Given its clinical importance, the accurate assessment of abdominal obesity is crucial. Waist circumference (WC) is the most widely used and accepted anthropometric parameter for directly quantifying central adiposity and visceral fat accumulation (8). Complementing this, Body Mass Index (BMI) serves as a standard measure for assessing overall body weight relative to height, providing a broader context of general adiposity (9). Together, these non-invasive measurements are fundamental tools in both clinical practice and epidemiological research for identifying individuals at high cardiometabolic risk.

The Mediterranean diet (MedDiet) has emerged as a cornerstone dietary pattern associated with numerous health benefits (10). Characterized by high consumption of fruits, vegetables, legumes, whole grains, nuts, fish, extra-virgin olive oil (EVOO), with moderate poultry intake and low red/processed meat and sweets consumption (11), the MedDiet has demonstrated efficacy in improving MetS components (12). EVOO itself represents the main source of fat in the MedDiet (13). It is constituted by a high content of monounsaturated fatty acids (MUFA), mainly oleic acid, a variable but significant amount of polyunsaturated fatty acids (PUFA), as well as minor amounts of antioxidant micronutrients like polyphenols, squalene, lignans, phenyl-ethyl alcohols and secoiridoids (14, 15). Oleic acid (18,1 n-9) accounts for approximately 49 to 83% of total fatty acids (FA) in EVOO (16), and its consumption has been linked to improved pancreatic and hepatic secretory activities, as well as reduced risk for development of gastric-duodenal ulcers (17). Previously, our research group has demonstrated the significant interaction of MUFAs with plasma lipids and lipoprotein compositions, ultimately resulting in reduced inflammation, coagulation and oxidative stress, and a significant improvement of glucose homeostasis and blood pressure (15, 18). These findings were further confirmed by health claims by the Food and Drug Administration (FDA) and the European Food Safety Authority (EFSA), which stated that oleic acid contained in EVOO, together with polyphenols, significantly contributes to the maintenance of normal blood cholesterol levels (19, 20). Beyond their effects on cholesterol, these bioactive polyphenols, such as hydroxytyrosol and oleocanthal, are known to exert potent anti-inflammatory and antioxidant effects by modulating key cellular signaling pathways involved in metabolic health. Experimental studies have demonstrated their ability to reduce markers of oxidative stress and inflammation, providing a strong mechanistic basis for the role of EVOO in preventing metabolic diseases linked to visceral adiposity (21, 22).

To build on the significant beneficial role of MedDiet and its key components on global health, our research group has validated the Chrono Med Diet Score (CMDS), an easy-to-use questionnaire, which considers lifestyle habits and a modern chrono-nutritional approach, in addition to information on food consumption. The CMDS consists of 11 food categories, and takes into account the time of farinaceous products intake and physical activity (23). EVOO is reputed a standalone factor, opposed to other sources of fat like butter or margarine. In previous studies, we highlighted that CMDS is associated not only with MedDiet adherence but also with several conditions, including abdominal obesity, dyslipidemia, glucose intolerance, increased cardiovascular risk, and gastrointestinal cancer (24).

Despite the recognized benefits of MedDiet and EVOO, gaps remain in understanding the specific contribution of EVOO consumption patterns within the context of overall chrono-dietary adherence. Specifically, the extent to which regular EVOO intake influences anthropometric outcomes like BMI and WC, independently or in conjunction with adherence to broader chrono-dietary principles (like CMDS), needs clarification. Furthermore, understanding the pathways through which these associations occur is crucial. In the present study, we aimed to investigate the associations between habitual EVOO intake frequency, adherence to the MedDiet, and key anthropometric measures in a large population sample.

## 2 Materials and methods

### 2.1 Study participants and data collection

The CMDS public survey was available online in both English and Italian for the general population since publication of the original study in April 2023 (25). The survey included some mandatory questions, such as age, height, weight. Following these inputs, participants could proceed with questions related to their dietary and lifestyle habits. Upon completion of the questionnaire, the system calculated an individual CMDS score and provided personalized health recommendations according to the results. No incentives were offered for the survey participation. Each participant was given a unique identifier based on internet protocol address, which was not collected, to avoid multiple inputs from the same subject in the online database. Only geographical area was registered for the purpose of the study, proving that 94.1% of the recorded input came from the European area. All responses were filled anonymously and on a voluntary basis. The survey was conducted in accordance with EU Regulation 2016/679 on the protection of natural persons about the processing of personal data (GDPR) (26). By completing the survey, all participants gave their written informed consent to participate in the present study. No personal data were stored following survey completion. The investigation was performed in accordance with the World Medical Association’s Declaration of Helsinki (27) and did not include any experiment involving human or biological human samples, nor research on identifiable human data. Since all responses were provided anonymously and on a voluntary basis, ethics committee approval was not required. Results were reported according to the Checklist for Reporting Results of Internet E-Surveys (CHERRIES) statement (28). The research study has been approved by Italian Ministry of University and Research (concession decree no. 1550 of October 11th, 2022) under the project PNRR-NRRP 2022; PE00000003 entitled “ON Foods - Research and innovation network on food and nutrition Sustainability, Safety and Security – Working ON Foods.”

### 2.2 Clinical and anthropometric assessment

Height was recorded in meters (m) and weight in kilograms (kg). BMI was calculated as weight divided by the square of height (kg/m^2^). Participants were categorized based on BMI as overweight (BMI 25.0–29.9 kg/m^2^) or obese (BMI ≥ 30.0 kg/m^2^) (29). For WC measurement, instructions taken from standard protocols were provided via the survey website; participants were advised to measure horizontally midway between the lowest rib margin and the superior iliac crest. Consistent with the study population being primarily European, abdominal obesity was defined according to the International Diabetes Federation (IDF) 2006 criteria for Europids as positive when above 94 cm in males and 80 cm in females (30, 31).

### 2.3 Chrono med diet score

The CMDS has been validated by our research group as a reliable score for evaluating adherence to the MedDiet, as well as its association with abdominal adiposity. Eleven food categories are evaluated to determine the overall score based on daily to weekly intake: (1) fruit, (2) vegetables and nuts, (3) legumes, (4) farinaceous products (i.e., bread, pasta, cookies), (5) grain cereals, (6) fish, (7) meat and meat products, (8) milk and dairy products, (9) EVOO, (10) butter, margarine and lard, and (11) alcohol intake. Two additional categories (time of farinaceous product intake and physical activity) were added to characterize the eating habits and overall metabolic health status. EVOO intake was assessed and categorized based on the frequency of consuming a reference amount, defined as approximately two tablespoons (25 grams) per day. Participants were classified into three groups. Sporadic intake was defined as consuming the reference amount of EVOO on fewer than 3 days per week. Frequent Intake was defined as consuming the reference amount on at least three but fewer than 6 days per week. Lastly, Regular Intake was defined as consuming the reference amount of EVOO on at least 6 days per week (23). The overall CMDS score ranged from −13 to 25 points, considering a cut-off of 13 points to discriminate MedDiet adherence (Supplementary Figure 1).

### 2.4 Statistical analysis

Descriptive statistical analyses of study sample were performed, and results were expressed as mean±standard deviation (SD). Comparisons of socio-demographic and anthropometric variables between two groups were conducted with the Student t-test for continuous variables. Comparisons between more than two groups were performed via one-way analysis of variance (ANOVA) followed by Bonferroni post-hoc test, where appropriate. To quantify the extent to which EVOO participated in the transmittance of change from adherence to the MedDiet to the increase in WC, a mediation analysis was performed. Furthermore, a logistic univariate model was tested to estimate the relationship between the exposure factor (EVOO) and the outcome of interest (abdominal obesity). Potentially confounding variables in the assessment of the causal effect were accounted for in a multivariable logistic regression. Results were expressed as Odds Ratio (OR) with their relative 95% confidence interval (95% CI) and graphically plotted in a forest plot. All statistical analyses were performed using the NCSS 2025 Statistical Software, version 25.0.2 (NCSS, LLC, Kaysville, Utah, United States) and GraphPad Prism, version 10.4.2 (GraphPad Software; San Diego, United States).

## 3 Results

### 3.1 Baseline characteristics of the study population

A total of 20,784 individuals were examined. Only subjects who declared to be 18 years old or older were included in the present study. Subsequently, 1,561 entries were removed due to incorrect or nonsensical data entry (e.g., text in numerical fields). An additional 1,671 participants were excluded based on outlier analysis, which identified clinically implausible anthropometric values. This resulted in a dataset of 17,552 potentially eligible participants. From this group, 1,279 individuals were excluded from the present analysis due to missing waist circumference data. Thus, the final analytical sample comprised 16,273 participants (7,561 males and 8,712 females). A flow diagram detailing participant selection is provided in Supplementary Figure 2.

Detailed baseline clinical characteristics stratified by sex are presented in Table 1. Males exhibited a slightly higher mean age (55.1 ± 11.6 years vs. 53.6 ± 13.1 years for females, *p* = 0.0316) and significantly higher mean BMI (26.5 ± 3.9 kg/m^2^ vs. 25.8 ± 3.1 kg/m^2^, *p* = 0.0192) and WC (94.5 ± 10.8 cm vs. 84.1 ± 7.5 cm, *p* < 0.001). Conversely, females showed significantly higher mean CMDS (12.9 ± 3.5 vs. 12.1 ± 4.2 for males, *p* = 0.0019). Abdominal obesity was prevalent in both sexes, identified in 60.6% of males and 65.3% of females. Differences in EVOO consumption frequency were also noted, with regular intake reported more often by females (30.8%) than males (23.9%).

**Table 1:** Clinical characterization of the study population divided by sex.

Clinical variable	Males	Females	*p*-value
*N*	7,561	8,712	
Age (years)			
Mean (SD)	55.1 (11.6)	53.6 (13.1)	0.0316*
Age ≥18 < 35 (*N*, %)	2,098 (27.7)	2,319 (26.6)	
Age ≥35 < 50 (*N*, %)	2,412 (31.9)	2,773 (31.8)	
Age ≥50 < 65 (*N*, %)	2,339 (30.9)	2,981 (34.2)	
Age ≥65 (N, %)	712 (9.4)	639 (7.3)	
BMI (kg/m^2^)			
Mean (SD)	26.5 (3.9)	25.8 (3.1)	0.0192*
BMI ≤ 18 (*N*, %)	312 (4.1)	499 (5.7)	
BMI ≥ 18 < 25 (*N*, %)	1,560 (20.6)	1,912 (21.9)	
BMI ≥ 25 < 30 (*N*, %)	3,578 (47.3)	3,910 (44.9)	
BMI ≥ 30 (*N*, %)	2,111 (27.9)	2,391 (27.4)	
WC (cm)			
Mean (SD)	94.5 (10.8)	84.1 (7.5)	0.0001***
Normal WC (*N*, %)	2,981 (39.4)	3,021 (34.7)	
Abdominal obese (*N*, %)	4,580 (60.6)	5,691 (65.3)	
CMDS			
Mean (SD)	12.1 (4.2)	12.9 (3.5)	0.0019**
CMDS ≤14 (*N*, %)	5,311 (70.2)	6,065 (69.6)	
CMDS >14 (*N*, %)	2,250 (29.8)	2,647 (30.4)	
EVOO intake			
Sporadic (*N*, %)	2,176 (28.8)	2,056 (23.6)	
Frequent (*N*, %)	3,578 (47.3)	3,971 (45.6)	
Regular (*N*, %)	1,807 (23.9)	2,685 (30.8)	

Comparisons were performed with Student t-Test. Data are presented as mean ± SD (standard deviation) or Number, Percentage (%). (*p < 0.05; ** *p* < 0.01; *** *p* < 0.001). N, Number; BMI, Body Mass Index; WC, Waist Circumference; CMDS, Chrono Med Diet Score; EVOO, Extra-Virgin Olive Oil.

### 3.2 Sporadic EVOO consumption identifies obese and younger subjects

To deepen our analysis on the potential role of EVOO in the definition of anthropometric measurements, we divided our population based on the referred EVOO intake into three categories (Table 2). First, we observed that individuals reporting regular EVOO consumption were significantly (overall ANOVA *p* = 0.0019, F-ratio = 14.4) older on average (55.9 ± 8.1) compared to both the sporadic (53.9 ± 7.1) and frequent intake groups (54.1 ± 7.7). Nevertheless, a significant inverse association was observed between EVOO intake frequency and BMI (overall ANOVA *p* = 0.0081, F-ratio = 7.8). Mean BMI in the regular intake group (24.7 ± 3.0) was significantly lower than both the frequent (25.9 ± 2.1) and sporadic intake groups (26.6 ± 2.9). This pattern was observed and significant within both male (*p* = 0.0029) and female subgroups (*p* < 0.001). Similarly, WC showed a significant inverse gradient with EVOO intake frequency (overall ANOVA *p* < 0.0001, F-ratio = 283.4). The regular intake group had the lowest mean WC (89.1 ± 6.7), significantly lower than the frequent (96.5 ± 8.3) and sporadic intake groups (99.4 ± 9.1). This association remained highly significant when analyzed separately for males (*p* < 0.0001) and females (*p* < 0.0001). Given the significant role of EVOO in the overall scoring system of the CMDS, we confirmed a strong positive association between EVOO intake frequency and CMDS adherence (overall ANOVA *p* < 0.0001, F-ratio = 503.2). Mean CMDS scores increased progressively as EVOO intake improved from sporadic (7.5 ± 2.1) to frequent (11.0 ± 1.9), finally to regular (13.9 ± 2.1), both in the overall population and when stratified by sex.

**Table 2:** Comparison of main clinical variables according to EVOO consumption.

Clinical variable	EVOO	EVOO	EVOO	*p*-value
	Sporadic Intake	Frequent Intake	Regular Intake	
N (M:F)	3,871 (2,112 M:1,759F)	7,791 (3,810 M:3,981F)	4,611 (1,639 M:2,972F)	
Age (years)				
Mean	53.9 ± 7.1^c^	54.1 ± 7.7^c^	55.9 ± 8.1^a,b^	0.0019**
Males	55.1 ± 7.2^c^	53.1 ± 5.9^c^	56.8 ± 8.9^a,b^	0.0381*
Females	52.4 ± 8.4	54.6 ± 6.5	54.9 ± 7.3	0.0822
BMI (kg/m^2^)				
Mean	26.6 ± 2.9^c^	25.9 ± 2.1^c^	24.7 ± 3.0^a,b^	0.0081**
Males	26.9 ± 2.1^b,c^	26.0 ± 2.5 ^a,c^	25.0 ± 2.1^a,b^	0.0029**
Females	26.1 ± 2.8^b,c^	25.5 ± 2.7^a,c^	24.2 ± 2.7^a,b^	0.0001***
WC (cm)				
Mean	99.4 ± 9.1^b,c^	96.5 ± 8.3^a,c^	89.1 ± 6.7^a,b^	0.0001***
Males	103.1 ± 7.8^b,c^	99.4 ± 9.3^a,c^	93.0 ± 3.1^a,b^	<0.0001***
Females	94.3 ± 6.3^b,c^	89.4 ± 7.3^a,c^	84.2 ± 5.5^a,b^	<0.0001***
CMDS				
Mean	7.5 ± 2.1^b,c^	11.0 ± 1.9^a,c^	13.9 ± 2.1^a,b^	<0.0001***
Males	7.9 ± 3.0^b,c^	10.4 ± 3.1^a,c^	12.8 ± 3.4^a,b^	<0.0001***
Females	6.7 ± 2.8^b,c^	12.7 ± 1.2^a,c^	14.8 ± 3.2^a,b^	<0.0001***

Comparisons were performed with ANOVA analysis. Data are presented as mean ± SD (standard deviation). ^a^Indicates significance with group with EVOO sporadic intake; ^b^Indicates significance with group with EVOO frequent intake; ^c^ Indicates significance with group with EVOO regular intake. (*p < 0.05; **p < 0.01; ***p < 0.001). EVOO, Extra-Virgin Olive Oil; BMI, Body Mass Index; WC, Waist Circumference; CMDS, Chrono Med Diet Score; N, Number; M, Males; F, Females.

### 3.3 EVOO intake as a mediator and independent factor for abdominal obesity

**Figure 1:**
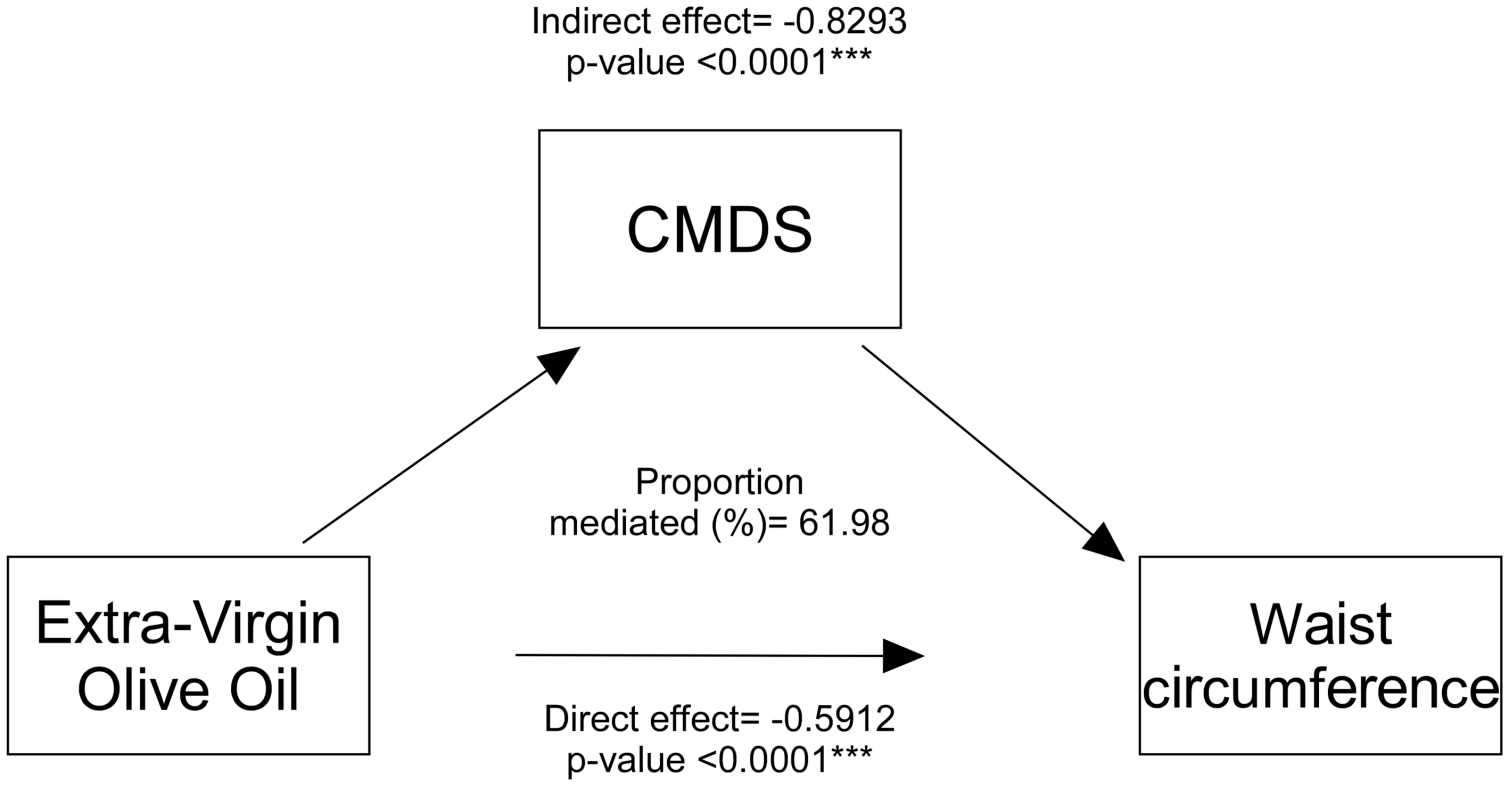
Mediation analysis of EVOO intake effect on waist circumference. Mediation model of the direct effect of Extra-Virgin Olive Oil (EVOO) intake on waist circumference and the indirect effect operating through the Chrono Med Diet Score (CMDS). Coefficients and *p*-values for direct and indirect paths are shown.

To further examine whether EVOO consumption could be considered a significant mediator in the relationship between CMDS adherence and WC, we performed a mediation analysis (Figure 1; Supplementary Table 1). We observed that EVOO intake indeed is a significant mediator (indirect effect coefficient = −0.8293, *p* < 0.0001), highlighting that part of the association between higher adherence to MedDiet and lower WC operates through EVOO intake. The analysis suggested this mediated pathway accounts for a substantial portion (61.98%) of the overall MedDiet-WC relationship. Furthermore, to determine if EVOO intake had an association with WC independent of its link with the CMDS, we looked at the direct effect of EVOO consumption on WC after controlling for CMDS. Results showed a highly significant direct effect (−0.5912, *p* < 0.0001), indicating that regular EVOO intake also was a standalone parameter in the definition of lower waist circumference, even when the overall CMDS score was accounted for.

### 3.4 Individuals with poor EVOO intake are at major risk of obesity

**Figure 2:**
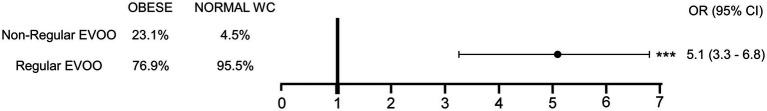
Risk for abdominal obesity according to EVOO intake after adjusting for potentially confounding variables. Contingency table to assess the Odds Ratio (OR) for abdominal obesity development according to EVOO intake and relative OR with 95% confidence interval is shown, following multivariable analysis to account for potential confounding variables. (***p < 0.0001). Extra-Virgin Olive Oil, EVOO; Waist Circumference, WC; Odds Ratio, OR; Confidence Interval, CI.

Based on the results of the mediation analysis, and given the significance of potentially confounding variables, we performed a logistic regression, adjusted for age, sex, and overall CMDS score, to analyze the risk for abdominal obesity based on EVOO intake patterns (Figure 2). Compared to individuals reporting regular EVOO intake, those with non-regular intake (sporadic and frequent combined) had significantly higher ORs of being classified as having abdominal obesity (OR = 5.1; 95% CI = 3.3–6.8; *p* < 0.0001).

## 4 Discussion

This large cross-sectional study provides compelling evidence linking regular EVOO consumption patterns with anthropometric outcomes. Our primary findings indicate that EVOO intake plays a significant role in the determination of WC, both as an independent factor and a mediator in the relationship between adherence to the MedDiet and anthropometric indices. Furthermore, our analysis suggests that non-regular EVOO consumption (2 tablespoons on fewer than 6 days per week) was associated with a striking five-fold increase in the odds of abdominal obesity, even after adjusting for potential confounders.

The observed inverse associations between EVOO intake frequency and anthropometric indices, especially WC, align with the well-documented benefits of the MedDiet, of which EVOO is a central component, on weight management and metabolic health. Indeed, it has been widely suggested that the consumption of a MedDiet rich in olive oil can prevent obesity (32, 33), type 2 diabetes mellitus (34), and MetS. Existing randomized clinical trials on the effects of EVOO on body weight and fat have yielded limited and conflicting results (35–37). Nonetheless, our research group has extensively studied in the last years the benefits of EVOO intake on the whole-body health, specifically analyzing the role of stearoyl-CoA desaturase 1 (SCD1), a crucial enzyme for the endogenous biosynthesis of oleic acid (C18:1, n9) and palmitoleic acid (C16:1, n7) from dietary or *de novo* synthesized saturated FA (15, 38, 39). Numerous studies have attempted to untangle the complex relationship between SCD1 and the progression of a variety of diseases, including obesity and diabetes (40), cardiovascular diseases (41, 42), and cancer (43). Previously, our group has studied the complex role of SCD1 in modulating hepatic lipid metabolism, inflammation, fibrosis, and susceptibility to liver diseases, highlighting the importance of intestinal MUFA production in maintaining hepatic health (44). Indeed, intestinal Scd1 deletion decreased hepatic MUFA levels, suggesting a direct contribution of gut-derived MUFAs to hepatic FA content. Nonetheless, we found that the absence of SCD1 and endogenously synthesized MUFAs in the intestinal epithelium increased susceptibility to inflammation and colorectal cancer development, which are reversed by dietary oleic acid supplementation (38). This could explain the magnitude of the differences observed between groups based on EVOO consumption, untangling the potential clinical relevance of habitual EVOO intake in relation to central adiposity, a key risk factor for cardiometabolic diseases. These results are in line with previously published data, highlighting the role of MedDiet and its components, especially EVOO, in reducing inflammation, improving lipid profiles, and enhancing insulin sensitivity, all of which can influence body composition (45). Indeed, in the European Prospective Investigation into Cancer and Nutrition (EPIC)-PANACEA prospective cohort, the high adherence to the MedDiet was linked to a decreased possibility to become overweight or obese (46). Moreover, Soriguer et al. followed-up 613 outpatients over 6 years and highlighted a significant decrease in obesity incidence in the group of patients who consumed EVOO instead of sunflower oil (47). Similarly, Guasch-Ferrè et al. found in two separate U. S. prospective cohorts that higher olive oil intake was associated with lower risk of total and cause-specific mortality, and that changing sources of fat from margarine, butter, mayonnaise, and dairy fat to EVOO was associated with lower risk of mortality (48). Our findings on better anthropometrics are part of a broader, though complex, picture of EVOO’s metabolic benefits. This is highlighted by a recent comprehensive meta-analysis of 33 randomized trials, which found that while EVOO consumption did not significantly alter most lipid profiles or blood pressure, it did lead to a significant improvement in insulin sensitivity. This specific effect on glucose metabolism and insulin resistance, which are central to the pathophysiology of abdominal obesity, provides a strong potential mechanism that could underlie the favorable body composition findings we observed in our cohort (49).

Moreover, bioactive polyphenols, such as hydroxytyrosol and oleocanthal, are known to exert potent anti-inflammatory and antioxidant effects by modulating key cellular signaling pathways involved in metabolic health. Experimental studies have demonstrated their ability to reduce markers of oxidative stress and inflammation, providing a strong mechanistic basis for the role of EVOO in preventing metabolic diseases linked to visceral adiposity (21, 22).

Nevertheless, the inherent overlap between MedDiet and EVOO challenging the isolation of EVO effects alone should be acknowledged. Anyhow, various findings suggest olive oil’s own positive impact on weight management. For instance, Buckland et al. demonstrated that regular EVOO use improves the taste of salads, vegetables, and legumes, thereby encouraging the consumption of high-fiber, low-energy-density foods that enhance fullness (50). From a biochemical perspective, the consumption of dietary oleic acid triggers satiety by acting as a molecular sensor that promotes the release of the gut-derived lipid messenger oleoylethanolamide (51). Nonetheless, in a randomized, double-blinded clinical trial by Galvão Cândido et al., fat loss was approximately 80% higher in women consuming an energy-restricted diet containing EVOO compared to a control group, proving again the role of EVOO in energy-restricted programs for obesity treatment (52).

The unique benefits of EVOO are further highlighted when compared to interventions with other vegetable oils, for which results are widely inconsistent. A comprehensive meta-analysis of 25 randomized controlled trials on canola oil found that its consumption led only to a modest decrease in overall body weight and had no significant effect on body fat markers (53). A randomized trial by Oliveira-de-Lira et al. in obese women on a calorie-restricted diet found that different oils produced distinct outcomes. While coconut oil supplementation led to greater reductions in waist circumference and body fat, chia oil was more effective at improving lipid profiles (54). Conversely, in a randomized controlled crossover study, coconut oil had no impact on anthropometric and biochemical findings (55). On this basis, one of the novel aspects of our study is the finding that EVOO is not only a component of diet that mediates a significant part of the MedDiet-WC relationship but can, indeed, be considered as a single active contributor to the benefits associated with MedDiet adherence on WC. The substantial OR linking non-regular EVOO intake to abdominal obesity further highlights the potential protective role of consistent EVOO consumption against this metabolically harmful fat accumulation.

To the best of our knowledge, this is the first study that provides novel evidence suggesting that EVOO can be considered a standalone, independent beneficial factor to WC. Its large sample size, which provides high statistical power to detect associations, is also a significant strength. Furthermore, it is one of the first studies to specifically categorize EVOO intake frequency and use robust statistical methods like mediation and multivariable logistic regression to isolate its independent effect within the context of a modern chrono-nutrition score. However, certain limitations must be acknowledged. The cross-sectional design prevents us from inferring causality between EVOO intake and lower abdominal obesity. Additionally, the reliance on self-reported data for both dietary intake and anthropometric measurements introduce potential for recall bias and measurement error. Finally, socio-economic data like annual income, marital status and smoking were missing from the online survey.

Despite these limitations, the findings have potential implications for public health and dietary guidelines. They support the recommendations emphasizing regular EVOO consumption within a healthy dietary pattern, such as the MedDiet, for maintaining a healthy weight and reducing central obesity. Future research should take advantage of longitudinal designs to establish temporality, utilize quantitative dietary assessment methods, include objective measures of adiposity and biomarkers, and ideally conduct randomized controlled trials to confirm the causal effects of regular EVOO intake on anthropometric outcomes and underlying metabolic pathways.

Taken all together, our findings on this large cross-sectional study demonstrate that regular EVOO consumption of is significantly associated with a more favorable anthropometric profile, characterized by lower BMI and WC, and greater adherence to a chrono-Mediterranean dietary pattern. EVOO intake plays a substantial role in the pathway linking adherence to this healthy dietary pattern and reduced central adiposity, while also indicating independent contributions from the broader dietary pattern. Conversely, non-regular EVOO consumption is associated with significantly increased odds of abdominal obesity. Future longitudinal and intervention studies are warranted to confirm these associations and elucidate the underlying mechanisms.

## Data Availability

The raw data supporting the conclusions of this article will be made available by the authors, without undue reservation.
