# Supplementary figures and images for: Regular extra-virgin olive oil intake independently associates with lower abdominal obesity
Source: Front Nutr. 2025 Sep 11;12:1645230. doi: 10.3389/fnut.2025.1645230 (PMC12461093; doi:10.3389/fnut.2025.1645230)

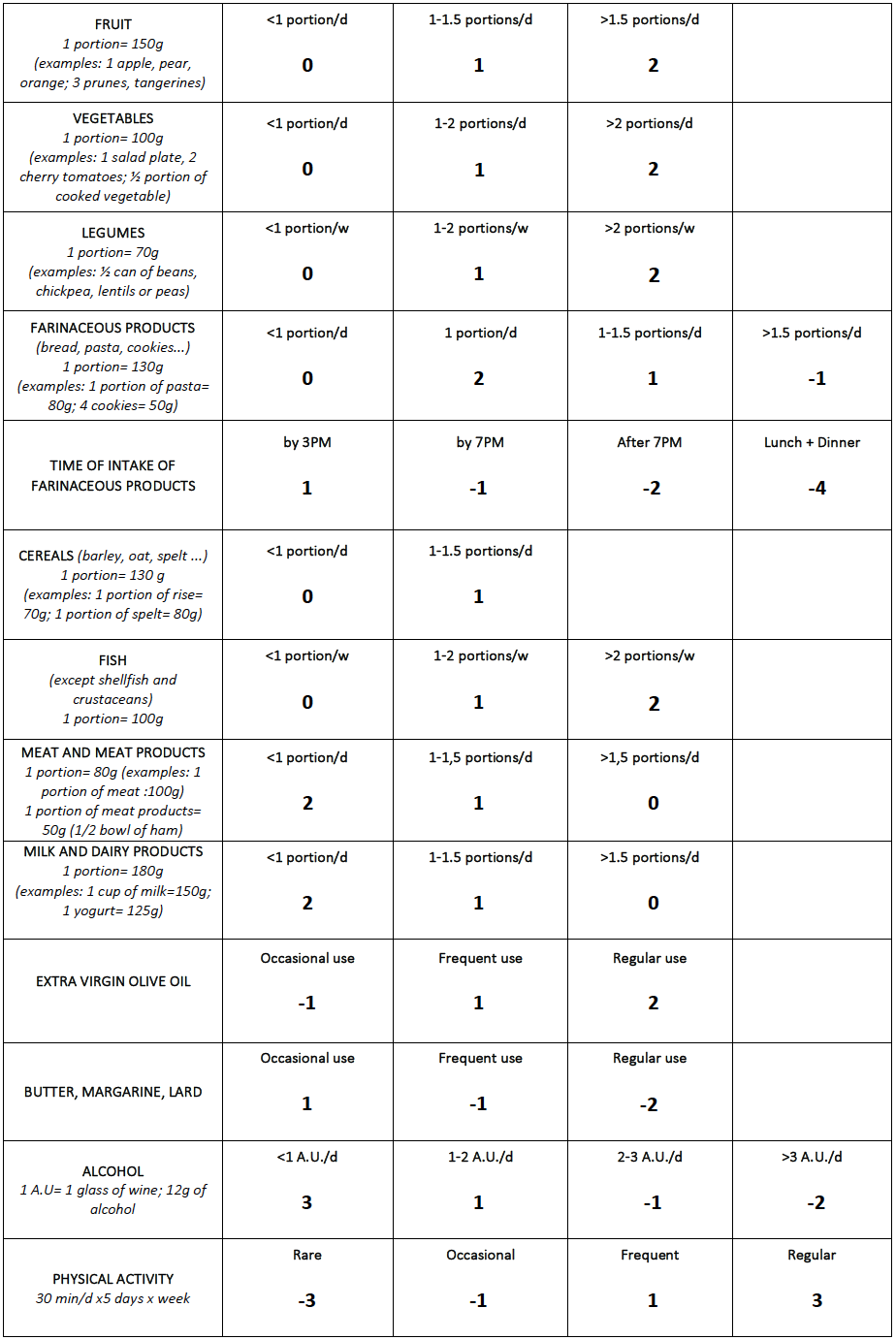

Supplement: Supplementary file 1 [file Image_1.PNG]

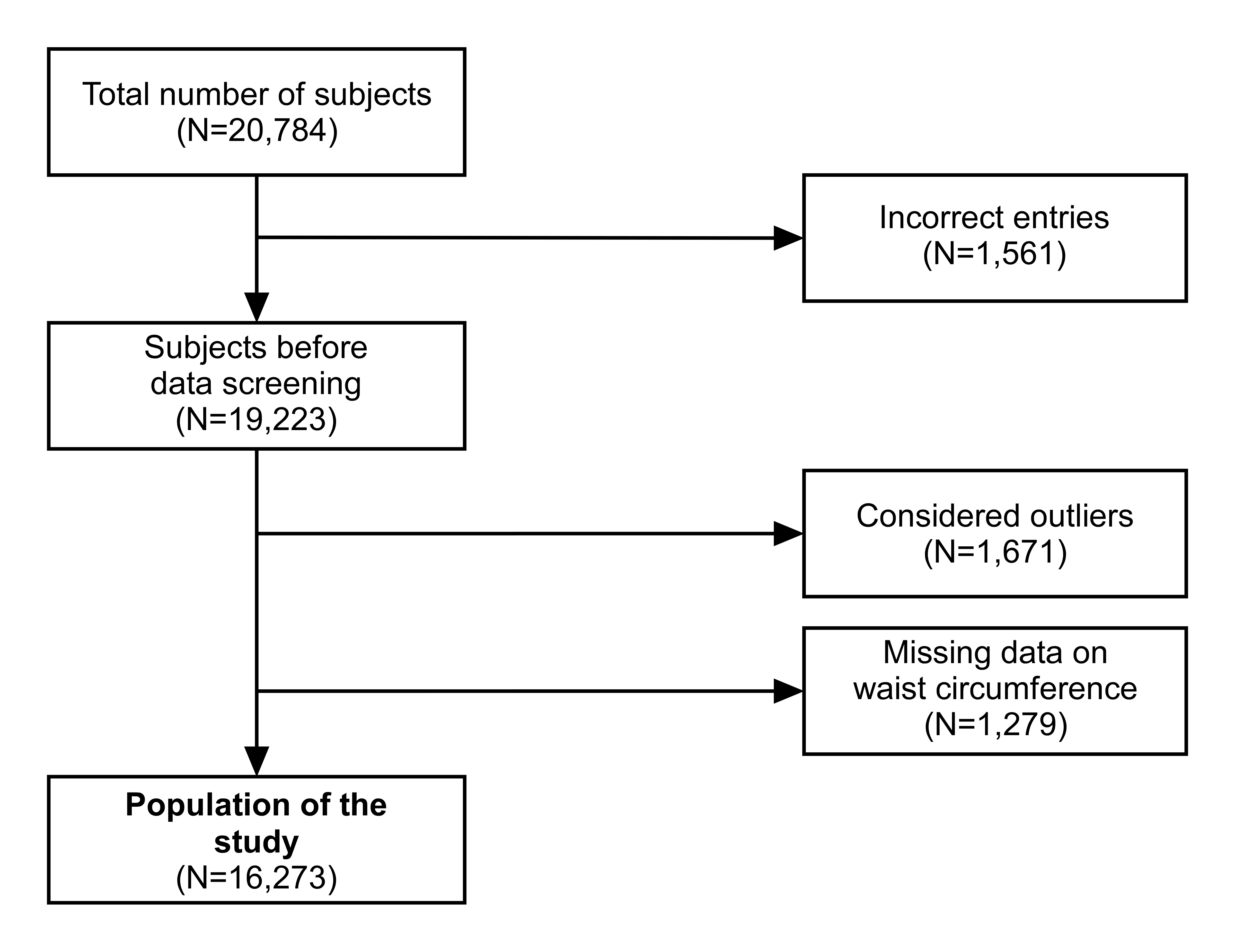

Supplement: Supplementary file 2 [file Image_2.TIF]
