# Supplementary material for: Regular extra-virgin olive oil intake independently associates with lower abdominal obesity
Source: Front Nutr. 2025 Sep 11;12:1645230. doi: 10.3389/fnut.2025.1645230 (PMC12461093; doi:10.3389/fnut.2025.1645230)
Supplement: Supplementary file 3 [file Supplementary_file_1.DOCX]

Supplementary Material

# Supplementary Tables

## Supplementary Table 1

**Glossary of Abbreviations**

| Abbreviation | Full word |
| --- | --- |
| ANOVA | Analysis of Variance |
| BMI | Body Mass Index |
| CHERRIES | Checklist for Reporting Results of Internet E-Surveys |
| CI | Confidence Interval |
| CMDS | Chrono Med Diet Score |
| EFSA | European Food Safety Authority |
| EPIC-PANACEA | European Prospective Investigation into Cancer and Nutrition–Physical Activity, Nutrition, Alcohol, Cessation of Smoking, Eating Out of Home and Obesity |
| EVOO | Extra-Virgin Olive Oil |
| FA | Fatty Acid |
| FDA | Food and Drug Administration |
| GDPR | General Data Protection Regulation |
| IDF | International Diabetes Federation |
| MedDiet | Mediterranean Diet |
| MetS | Metabolic Syndrome |
| MUFA | Monounsaturated Fatty Acids |
| OR | Odds Ratio |
| PUFA | Polyunsaturated Fatty Acids |
| SCD1 | Stearoyl-CoA Desaturase 1 |
| SD | Standard Deviation |
| SE | Standard Error |
| WC | Waist Circumference |

## Supplementary Table 2

| Variable | β Coefficient | SE | 95% CI for β | OR | 95% CI for OR | p-value |
| --- | --- | --- | --- | --- | --- | --- |
| EVOO | 1.63 | 0.19 | 1.19 – 1.92 | 5.1 | 3.3 – 6.8 | <0.0001 |
| Age | 0.03 | 0.008 | 0.01 – 0.03 | 1.02 | 1.01 – 1.04 | <0.001 |
| Sex | -0.22 | 0.1 | -0.38 – -0.06 | 0.8 | 0.68 – 0.94 | <0.01 |
| CMDS | -0.16 | 0.08 | -0.18 – -0.14 | 0.85 | 0.83 – 0.95 | <0.0001 |

Model Fit: Nagelkerke R² = 0.26; AUC = 0.86.

**Logistic regression for risk for abdominal obesity according to EVOO intake after adjusting for potentially confounding variables.** Abbreviations: Standard Error, SE; Confidence Interval, CI; Odds Ratio, OR; Extra-Virgin Olive Oil, EVOO; Chrono Med Diet Score, CMDS; Area Under the Curve, AUC.
